# Sequencing of idiopathic pulmonary fibrosis-related genes reveals independent single gene associations

**DOI:** 10.1136/bmjresp-2014-000057

**Published:** 2014-12-10

**Authors:** Meghan A Coghlan, Adrian Shifren, Howard J Huang, Tonya D Russell, Robi D Mitra, Qunyuan Zhang, Daniel J Wegner, F Sessions Cole, Aaron Hamvas

**Affiliations:** 1 Division of NewbornMedicine, EdwardMallinckrodt Department of Pediatrics, Washington University School of Medicine, St. Louis, Missouri, USA; 2 Division of Pulmonary and Critical Care Medicine, Department of Internal Medicine, Washington University School of Medicine, St. Louis, Missouri, USA; 3 Department of Genetics, Center for Genome Sciences and Systems Biology, Washington University School of Medicine, St. Louis, Missouri, USA; 4 Division of Statistical Genomics, Department of Genetics, Washington University School of Medicine, St. Louis, Missouri, USA; 5 Division of Neonatology, Department of Pediatrics, Ann and Robert H. Lurie Children's Hospital of Chicago, Northwestern University Feinberg School of Medicine, Chicago, Illinois, USA

**Keywords:** Interstitial Fibrosis, Paediatric Lung Disaese, Rare lung diseases, COPD epidemiology

## Abstract

**Background:**

Previous studies investigating a genetic basis for idiopathic pulmonary fibrosis (IPF) have focused on resequencing single genes in IPF kindreds or cohorts to determine the genetic contributions to IPF. None has investigated interactions among the candidate genes.

**Objective:**

To compare the frequencies and interactions of mutations in six IPF-associated genes in a cohort of 132 individuals with IPF with those of a disease-control cohort of 192 individuals with chronic obstructive pulmonary disease (COPD) and the population represented in the Exome Variant Server.

**Methods:**

We resequenced the genes encoding surfactant proteins A2 (*SFTPA2*), and C (*SFTPC*), the ATP binding cassette member A3 (*ABCA3*), telomerase (*TERT*), thyroid transcription factor (*NKX2-1*) and mucin *5B* (*MUC5B*) and compared the collapsed frequencies of rare (minor allele frequency <1%), computationally predicted deleterious variants in each cohort. We also genotyped a common *MUC5B* promoter variant that is over-represented in individuals with IPF.

**Results:**

We found 15 mutations in 14 individuals (11%) in the IPF cohort: (*SFTPA2* (n=1), *SFTPC* (n=5), *ABCA3* (n=4) and *TERT* (n=5)). No individual with IPF had two different mutations, but one individual with IPF was homozygous for p.E292V, the most common *ABCA3* disease-causing variant. We did not detect an interaction between any of the mutations and the *MUC5B* promoter variant.

**Conclusions:**

Rare mutations in *SFTPA2*, *SFTPC* and *TERT* are collectively over-represented in individuals with IPF. Genetic analysis and counselling should be considered as part of the IPF evaluation.

**Key messages**

- Rare mutations in *SFTPA2*, *SFTPC,* (surfactant proteins) and *TERT* (telomerase) were found in individuals with idiopathic pulmonary fibrosis (IPF) but not those with chronic obstructive pulmonary disease.
- Mutations associated with childhood interstitial lung disease are present in adults with IPF supporting that these mutations predispose to a spectrum of interstitial lung diseases manifesting at all ages.
- Genetic analysis and counselling should be considered as part of the IPF evaluation.

## Introduction

Idiopathic pulmonary fibrosis (IPF) is the most common chronic interstitial lung disease (ILD) in adults.^1^ With an average presenting age of 60–70 years, the clinical course is one of progressive decline in lung function or precipitous deterioration following an acute exacerbation. With a median survival of only 2–3 years after diagnosis, it is one of the most common indications for lung transplantation in adults.^1^ ^2^ Despite an association with smoking and environmental exposures, the pathogenesis of this disease remains elusive.^1^ Familial IPF, defined by disease in two or more first-degree relatives, is estimated to occur in 2–20% of all IPF cases and can present at earlier ages.^1^ ^3^

Previous studies investigating genetic predisposition in sporadic and familial pulmonary fibrosis have identified rare mutations in the gene encoding telomerase (*TERT,* Gene ID 7015) and in the surfactant-associated genes, surfactant protein A2 (*SFTPA2,* Gene ID 729238), surfactant protein C (*SFTPC,* Gene ID 6440) and the ATP-binding cassette member A3 (*ABCA3,* Gene ID 21).^4–11^ Additionally, a polymorphism (rs35705950) in the mucin 5B gene (*MUC5B,* Gene ID 727897) promoter is significantly more prevalent in individuals with both sporadic and familial IPF.^12^ ^13^ Mutations in *SFTPC, ABCA3,* and the thyroid transcription factor gene (*NKX2-1,* Gene ID 7080) are associated with childhood ILD.^9^ ^14–19^ In studies of individuals and families with mutations in *SFTPC*, disease penetrance varies in severity, age of onset, histopathology and clinical characteristics suggesting that childhood ILD and IPF in adulthood may be part of the same spectrum of disease.^9^ ^10^ ^18–21^

Since no previous studies investigated all of these candidate genes simultaneously in IPF, we resequenced these genes in a cohort of patients with IPF to test the hypothesis that mutations in the coding regions of these six genes occur at a higher frequency in IPF than in chronic obstructive pulmonary disease (COPD) or in population-based cohorts, and to determine if interactions among these genes and the *MUC5B* promoter polymorphism modified disease phenotype.

## Methods

### Subject selection

#### IPF cohort

Individuals with IPF were included if they had a usual interstitial pneumonia (UIP) pattern on lung explant or biopsy, or had a clinical diagnosis of IPF validated by the research team using American Thoracic Society/European Respiratory Society 2011 criteria.^1^ Individuals with non-IPF/UIP idiopathic interstitial pneumonia, hypersensitivity pneumonitis, occupational lung disease, drug-induced lung disease and connective tissue disorders were excluded. Recruitment included all individuals with IPF regardless of race, gender, or age presenting from 2009 to 2012. This cohort included archived DNA samples acquired through a waiver of consent from patients in the Washington University/Barnes-Jewish Hospital Adult Lung Transplant Programme who underwent lung transplantation for IPF (n=105) and prospectively recruited individuals followed in the Division of Pulmonary and Critical Care Medicine (n=27) for a total n=132, which was a sample size of convenience. Clinical data, including demographic information, age at onset of symptoms and diagnosis, family history, smoking history, exposure history and results from radiological studies and lung biopsies, were acquired through medical record review.

#### COPD cohort

Archived DNA samples from individuals with COPD through the COPD SCCOR Project at Washington University/Barnes-Jewish Hospital (5 P50 HL084922) from patients recruited from 2005 to 2011 were used as a disease-based control. Through permission for sample sharing, we obtained the DNA and linked de-identified clinical information including age, gender, race and smoking history from existing databases through a waiver of consent. The clinical characteristics of each cohort are summarised in table 1.

**Table 1:** Characteristics of the IPF and COPD cohorts

	IPF (n=132)	COPD (n=192)	p Value (IPF vs COPD)
Age	54.6±9.2	60.2±8.1	0.5
Gender			
Female	34 (26%)	93 (48%)	
Male	98 (74%)	99 (52%)	<0.001
Race			
Caucasian	119 (90%)	179 (93%)	0.43
African–American	6 (5%)	10 (5%)	
Asian	4 (3%)	0 (0%)	
Hispanic	3 (2%)	2 (1%)	
Multiple races	0 (0%)	1 (0.5%)	
Tobacco use			
Yes	84 (64%)	189 (98%)	
No	47 (36%)	2 (1%)	<0.001
Unknown	1 (1%)	1 (0.5%)	

COPD, chronic obstructive pulmonary disease; IPF, idiopathic pulmonary fibrosis.

#### Exome sequencing project database

Data from the NHLBI Exome Sequencing Project (ESP, version ESP6500SI-V2, http://evs.gs.washington.edu/EVS/) were used to provide race-stratified, population-based frequencies of variants in candidate genes (accessed 1 July 2013).

### Gene selection

Based on our hypothesis that childhood ILD and IPF may be part of a spectrum of disease, we selected genes that were known at the time we designed the study between associate with IPF only (*SFTPA2* and *TERT*) and genes associated with both childhood ILD and IPF (*ABCA3*, *SFTPC* and *NKX2-1*). Mutations in *ABCA3* are expressed in a recessive manner, while mutations in *SFTPA2*, *SFTPC, NKX2-1 and TERT* are dominantly expressed. The presence of either one or two copies of the *MUC5B* promoter variant is associated with IPF, however, exonic mutations in *MUC5B* have not been studied. Therefore, we also interrogated *MUC5B*, though the mode of inheritance is unknown. Although mutations in the telomerase RNA component gene (*TERC*, Gene ID 7012) are also associated with IPF, the mutations occur in the part of the gene that encodes the RNA component and we therefore could not apply our definition of ‘mutation’ based on alteration in protein function.^6^ ^22^ ^23^

### Sample preparation and sequencing

We performed DNA purification on preserved saliva samples obtained from the patients with IPF using Oragene DNA self-collection kits and purification protocol (DNA Genotek Inc, Kanata, Ontario, Canada). The DNA from each specimen was quantified using the Qubit Fluorometer and equimolar amounts of each patient's DNA were pooled. Using this pooled DNA, all exons of *ABCA3, MUC5B* (except exon 49 due to its large size (∼11 kb) and the presence of significant repetitive sequence that prevented standard sequencing approach), *NKX2-1, SFTPA2, SFTPC* and *TERT* were amplified*,* approximately 45 kb of sequence per individual. Equal amounts of amplified product were pooled for next generation sequencing using the Illumina Genome Analyzer/MiSeq platform. Negative and positive controls were inserted into the pooled sample to optimise detection of rare variants with high specificity and sensitivity.^24^ ^25^

We used SPLINTER (Short IN/DEL Prediction by Large deviation Inference and Non-linear True frequency Estimation by Recursion) to identify rare single nucleotide variants and small insertions/deletions.^25^ We defined a mutation as a small insertion or deletion or a non-synonymous single nucleotide variant previously identified in children or adults with respiratory disease and/or predicted to disrupt encoded protein function in at least 2 of 3 prediction algorithms: SIFT, PolyPhen2, or Mutation Taster.^26–28^ Since IPF is a rare disease, we excluded variants with a prevalence of >1% in the general population as catalogued in the NHLBI Exome Sequencing Project (ESP, version ESP6500SI-V2, http://evs.gs.washington.edu/EVS/ (accessed July 1, 2013)). We then validated using an independent genotyping strategy through Sanger resequencing or Taqman Genotyping Assays. Taqman genotyping was also used for the *MUC5B* promoter polymorphism. The validation of variants allowed us to confirm the frequency of each variant and to link it to an individual.

### Data analysis

Study data were collected and managed using REDCap electronic data capture tools hosted at Washington University School of Medicine's Institute for Clinical and Translational Studies.^29^ Since it is unlikely that an individual carries more than one rare mutation at a single gene locus, the number of mutations in a single gene can be collapsed for statistical purposes and compared using a univariate test.^30^ We used Fisher's Exact tests to compare frequencies of individual variants and collapsed frequencies across a gene, and to test the difference of other categorical features (gender, race, tobacco use). We used t tests to test the difference in age of onset between mutated and non-mutated IPF samples.

## Results

### Mutations in IPF-associated and childhood ILD-associated genes

In the IPF population, we found 15 mutations in 14 different individuals in all the genes interrogated except *NKX2-1*. There were no differences in age of onset, sex or tobacco use in the IPF individuals with or without a mutation (table 2). No individuals had single rare mutations in two different genes, although one individual was homozygous for *ABCA3* p.E292V. There was one mutation found in *SFTPA2* in the IPF cohort and none in the COPD cohort but the collapsed frequency was not statistically significant (table 3).

**Table 2:** Characteristics of IPF individuals with and without mutations

	IPF		
	Mutation (n=14)	Number of mutation (n=118)	p Value
Age	49.6±14	55.2±8.4	0.51
Gender			
Female	3 (21%)	31 (26%)	1.0
Male	11 (79%)	87 (74%)	
Tobacco use			
Yes	8 (57%)	76 (64%)	0.57
No	6 (43%)	41 (35%)	
Unknown	0 (0%)	1 (1%)	
Outcome			
Alive w/o lung transplant	3 (21%)	20 (17%)	
Lung transplant*	11 (79%)	95 (81%)	
Died w/o lung transplant	0 (0%)	3 (3%)	

*Individuals who had a transplant and then died are included in the ‘transplant’ numbers.

IPF, idiopathic pulmonary fibrosis.

**Table 3:** Mutations identified in IPF and COPD cohorts of European descent

Gene	Mutation	Amino acid change	IPF* (ED) (n=119) (n (MAF))	COPD (n=178) (n (MAF))	ESP (ED) (MAF), %	p Value (IPF vs COPD)	dbSNP number
*SFTPA2*	c.532G>A	V178M	1 (0.4%)†	0	0.01	0.4	
*SFTPC*	c.218T>C	I73T	3 (1.3%)†	0	0		rs121917834
	c.329T>G	L110R	1 (0.4%)	0	0		
	c.334G>A	A112T	1 (0.4%)	0	0		
Collapsed frequency			2.1%			0.01	

*ABCA3*	c.875A>T	E292V	4 (1.68%)	3 (0.84%)	0.45		rs149989682
	c.4420G>A	R1474W	0	3 (0.84%)	0.36		rs146709251
Collapsed frequency			1.68%	1.68%		1.0	

*NKX2-1*			0	0	NA		
*TERT*	c.323G>C	‡R108P	1 (0.42%)	0	0		
	c.994C>T	‡L332F	1 (0.42%)	0	0		
	c.1775A>G	‡H592R	1 (0.42%)†	0	0		
	c.2110C>T	P704S	2 (0.84%)	0	0		rs199422297
Collapsed frequency			2.1%			0.01	

*All individuals with mutations were of European descent.

†One individual each with a family history.

‡Novel mutations.

ED, European descent; MAF, minor allele frequency.

All mutations in IPF and COPD cohorts were found only in individuals of European descent, so the analyses in table 3 focused on this subset. Mutations in *SFTPC* were found only in the IPF cohort and included the common disease-causing allele p.I73T as well as two other mutations (p.L110R and p.A112T) previously identified in children with ILD but not in adults with IPF (table 3, unpublished data A Hamvas and L M Nogee, 2014).^15^ None of the *SFTPC* mutations was present in individuals with COPD or in the ESP database. The collapsed frequency of *SFTPC* mutations in the IPF cohort was statistically significant when compared to the COPD cohort.

Rare mutations in *TERT* were also present in the IPF cohort and not found in COPD. The collapsed frequency of *TERT* mutations in the IPF cohort was statistically greater than that of the COPD cohort (table 3).

To determine if the individuals in the IPF and COPD cohorts were representative of the general population, we also examined the frequencies of common variants in these genes. Although none of these common variants are predicted to be deleterious, we cannot exclude the possibility that some of these variants could be in linkage with our functional mutations, but our sequencing methodology did not permit identifying on which allele these variants resided. The common non-synonymous variants in *SFTPC* (p.T138N and p.S186N) and *SFTPA2* (p.A91P) were present in similar frequencies in the IPF and COPD cohorts compared to the general population, as were the common synonymous variants in *ABCA3* (p.F353F, p.P585P and p.S1372S) (see online supplementary table S1). The frequency of *SFTPA2* p.Q223K was less than the ESP frequency, which could be due to sample size. The frequencies of the common *TERT* variant p.A279T, previously associated with aplastic anaemia, were similar in IPF and COPD cohorts and lower than the 3% population-based frequency in the ESP, suggesting that this mutation is not contributing to pulmonary disease (see online supplementary table S1).^18^

### Familial IPF

Of the 132 individuals in the IPF cohort, 26 (20%) reported a family history of IPF. In those 26 individuals, only 3 (12%) had a mutation identified in one of the 6 genes, one each in *SFTPA2*, *SFTPC* and *TERT*. The remaining 23 individuals (88%) did not have a mutation in any of these candidate genes that explained their family history.

Interestingly, the other 11 individuals in the IPF cohort with an identified mutation did not have a family history of IPF, suggesting (1) that the mutations were spontaneous in these individuals, (2) the mutations were inherited but not penetrant in the parent, (3) in the case of novel mutations, they were not disease causing or (4) family history of pulmonary fibrosis had not been fully ascertained or recognised through family member recall.

### Mutations in *MUC5B*

Collapsed frequencies of rare coding region mutations in *MUC5B* were similar in the IPF and COPD cohorts and in the ESP database. Consequently, mutations in the coding sequence did not appear to be associated with attributable risk for IPF and were analysed separately from mutations in the other genes (table 4). Interestingly, p.V5436M was significantly under-represented in the IPF cohort (p=0.002), but the clinical significance, if any, of this observation is unclear (see online supplementary table S1).

**Table 4:** *MUC5B* mutations in individuals of European descent

Gene	Mutation	Amino acid prediction	IPF (n=119) (n (MAF))	COPD (n=178) (n (MAF))	ESP (ED) (MAF), %	dbSNP number
*MUC5B*	c.356G>A	R119H	0	2 (0.6%)	0.03	rs199733278
	c.1520C>T	T507M	1 (0.4%)	0	0.09	rs201605309
	c.1855C>T	R619W	2 (0.8%)	2 (0.5%)	0.38	rs56411917
	c.1994C>T	*A665V	1 (0.4%)	0		
	c.14896G>A	G4966S	0	3 (0.8%)	0.3	rs56217440
	Collapsed freq		1.7%	2.0%		

*Novel mutation.

ED, European descent; IPF, idiopathic pulmonary fibrosis; MAF, minor allele frequency.

We also tested both cohorts for the previously reported IPF-associated *MUC5B* promoter polymorphism (rs35705950; table 5). The 28% minor allele frequency (MAF) in our IPF cohort is significantly greater than the MAF in either the COPD cohort (13%, p<0.001) or the previously reported population-based frequency of 9%, p<0.001.^12^ ^31^ The similar prevalence of the rs35705950 polymorphism in individuals with or without mutations in the other six genes studied in either cohort (p=1.0) suggests that the polymorphism does not interact with mutations in these genes to modify disease penetrance (table 5, online supplementary table S2). However, the small sample size of this study limits the ability to truly detect interactions.

**Table 5:** *MUC5B* promoter variant (rs35705950) genotypes and minor allele frequencies (MAF) in individuals with IPF-associated mutations

	IPF			COPD		
	Mutation (n=14)	No Mutation (n=118)	Total (n=132)	Mutation (n=6)	No Mutation (n=186)	Total (n=192)
G/G	6 (43%)	57 (48%)	63 (48%)	6 (100%)	140 (75%)	146 (76%)
G/T	8 (57%)	57 (48%)	65 (49%)	0 (0%)	44 (24%)	44 (23%)
T/T	0 (0%)	4 (3%)	4 (3%)	0 (0%)	2 (1%)	2 (1%)
MAF	8 (29%)*	65 (28%)*	73 (28%)†	0 (0%)	48 (13%)	48 (13%)†

p Value IPF versus population (MAF 9%)=6.7 e-12; COPD versus population=0.09.^12^

p Value: COPD no mutation versus mutation=0.38.

*p Value IPF mutation versus no mutation=1.0.

†p Value of IPF versus COPD=1.9 e-06.

COPD, chronic obstructive pulmonary disease; IPF, idiopathic pulmonary fibrosis.

## Discussion

We demonstrated that previously described rare, dominantly expressed mutations in *SFTPC*, *SFTPA2* and *TERT* occur in patients with sporadic and familial IPF.^4–10^ However, our study is the first in which all genes associated with both IPF and/or childhood ILD were resequenced simultaneously in a cohort of individuals with both sporadic and familial IPF. The lack of mutations in more than one gene in each individual suggests that interactions among these genes do not contribute significantly to disease expression, although significantly larger cohorts are necessary to confirm this preliminary observation and possibly identify further rare variants. Furthermore, recently published genome-wide association studies have identified other candidate loci associated with increased risk of IPF and should be interrogated in future studies but had not been identified at the time that gene selection for this study occurred.^13^ ^32^

In this study, 14 (11%) of the individuals in the IPF cohort had mutations in IPF-associated genes. This frequency may be either overestimated or underestimated based on our definition of a ‘mutation’ which relied on a previous association of the gene with disease, or computationally predicted effects on protein function. We used a stringent definition for altered function by requiring that 2 of 3 algorithms predicted functionality. However, only testing in a relevant model system and replication in an independent cohort can confirm the functional significance of these mutations. Additionally, our IPF cohort was weighted toward individuals who were referred for lung transplantation which could bias towards younger individuals, and thus, could also be enriching the cohort for genetically-based disease. The significant gender difference between the IPF and COPD cohorts is consistent with the demographics of each disease and mutations were identified in individuals of both genders in each cohort but any possible gender affect on rare variant discovery would have to be investigated with a larger patient population.

### IPF and childhood ILD-associated genes

#### Surfactant-associated genes

As anticipated from other studies, we found mutations in *SFTPC* in one individual with familial IPF (4%) and four individuals with sporadic IPF (4%). Previous studies have demonstrated the prevalence of *SFTPC* mutations to be as high as 25% in familial pulmonary fibrosis (without a distinction from IPF, specifically) and as low as 0.7% in sporadic IPF.^7^ ^8^ The *SFTPC* mutations identified in our cohort have previously been identified in children and adults with ILD and/or IPF. Two of these mutations, p.I73T and p.L110R, result in aberrant prosurfactant protein C protein products in vitro.^33^ Since we did not find any mutations in *SFTPC* in the COPD cohort, and they are not present in the general population, this enrichment in the IPF cohort further strengthens the evidence that these dominantly expressed mutations are disease-causing and demonstrates that even ‘sporadic’ IPF may have a genetic basis due to spontaneous mutation.

Dominant mutations in *SFTPA2* have been identified in kindreds with familial IPF and lung cancer.^4^ In our study, p.V178M was present in one individual with familial IPF and was seen only once in ESP. However, no other affected family members in this kindred were available to determine if this mutation segregates with disease, so it is difficult to determine if this mutation is disease-causing.

Homozygous recessive or compound heterozygous *ABCA3* mutations cause neonatal respiratory failure and childhood ILD, and single *ABCA3* mutations (p.E292V and p.D123N) interacting with *SFTPC* p.I73T have been reported in two families, one with childhood ILD and one with IPF.^10^ ^34^ Our identification of one individual who was homozygous for *ABCA3* p.E292V adds to the recent identification of a kindred with pulmonary fibrosis due to a p.G964D thus further supporting the possibility that adult-onset fibrotic lung disease due to homozygous or compound heterozygous mutations in *ABCA3* may occur.^11^ In contrast to our previous study that demonstrated an enrichment of single *ABCA3* mutations in newborns with RDS, suggesting a developmental interaction that increased risk or severity of disease, we did not find the frequency of single *ABCA3* mutations in the IPF cohort to be greater than the 3–5% in the general population. This is consistent with a prior study showing single E292V mutations were not a major risk factor for pulmonary disease in the general population, and also suggests that interactions with other modifiers are less likely to increase risk for disease.^35–37^

Since mutations in *NKX2-1* are extremely rare in the general population, the lack of identifiable mutations in *NKX2-1* in our IPF and COPD cohorts may simply be a function of the limited sample size of our cohort, thus making it difficult to know the true prevalence of *NKX2-1* mutations in patients with IPF.

### TERT

Previous studies of *TERT* and the RNA component of telomerase (*TERC*) have shown heterozygous mutations to be present in 8–15% of familial IPF cohorts.^6^ In our study population, novel mutations in *TERT* were only seen in the IPF cohort, solidifying the link between this gene and predisposition to IPF. That the p.A279T mutation was present in IPF and COPD cohorts with similar frequencies makes it unlikely that it contributes directly to the pathogenesis of IPF. Complex functional assays will be necessary to identify any mechanisms whereby these mutations exert an effect.

### MUC5B

Since the rs35705950 promoter polymorphism is over-represented in individuals with IPF, we resequenced all but the largest exon of *MUC5B* to determine if mutations in the coding sequence of the gene might also associate with IPF. Although we found individual rare deleterious variants in IPF and COPD cohorts, the similar collapsed frequencies in both cohorts makes it unlikely that mutations in coding regions of this gene contribute appreciably to the risk of IPF.

The MAF of the *MUC5B* promoter polymorphism was approximately 30% in our IPF cohort which is similar to previous reports.^12^ ^38^ Since this frequency is similar in patients with IPF with and without other candidate gene mutations, this polymorphism is not associated with increased risk for disease in association with the exonic mutations in *ABCA3*, *SFTPA2*, *SFTPC* or *TERT*.

## Conclusion

The presence of childhood ILD-associated gene mutations in adults with IPF supports the hypothesis that these mutations predispose to a spectrum of fibrotic lung diseases manifesting from infancy to adulthood. Therefore, it is possible that childhood ILD and adult IPF represent variable expression of a common underlying gene-based pathogenic mechanism. While these mutations increase disease risk, the environmental modifiers that cause injury, disrupt repair and ultimately result in disease remain to be identified, along with the full complement of gene mutations predisposing to fibrotic lung disease. While possible that exome sequencing may only capture coding region variants and disregard non-coding elements of genome leading to an underestimate the genetic contribution of a gene to IPF, further investigation of genetic susceptibility to IPF using exome sequencing of affected and unaffected family members could elucidate not only the relationship between childhood ILD and adult IPF, but also the unidentified genetic factors leading to disease expression.
